# Telling tales: unlocking the potential of AAC technologies

**DOI:** 10.1111/1460-6984.12449

**Published:** 2018-12-30

**Authors:** Annalu Waller

**Affiliations:** ^1^ Computing, School of Science and Engineering University of Dundee Dundee UK

## Abstract

Augmentative and alternative communication (AAC) has been transformed by the social media revolution made possible by the emergence of mobile technology. The cumbersome dedicated devices of the 1970s have evolved into a burgeoning AAC app industry. However, the limited use and abandonment of AAC technologies remains high. Unlocking the untapped potential of technology requires a paradigm shift in the design of AAC technologies by building systems that minimize the cognitive load placed on users, adapting to their individual physical and language needs. Telling Tales shares insights and stories of how the combination of user‐centred design, interdisciplinary research and the application of intelligent computing is providing a vision of future generations of AAC technologies.

## Introduction

It is an honour to have been invited to deliver the 2017/18 Winter Lecture for the IJLCD to an audience comprised mostly of speech and language therapists (SLTs). The honour is more poignant considering my life‐long relationship with SLTs, first as a child growing up with dysarthric speech due to cerebral palsy and latterly as a rehabilitation engineer working in the field of augmentative and alternative communication (AAC). My interest in AAC was sparked when, during the third year of my Computer Science degree, *Byte* magazine (1982) published a special issue on ‘Computers and the Disabled’ in which early speech‐generating devices (SGDs) were featured.

The advent of microcomputing in the 1970s and the subsequent development of computer‐based assistive technologies (ATs) opened up unimagined opportunities for people with speech and/or physical impairments. Prior to electronics, early communication technologies enabled people to create written text using mechanical or electric solutions. Mechanical systems which gave access to typing for people with no hand function, such as the prototype POSM sip‐and‐puff electric typewriter in 1960, were superseded by transistor‐based systems in the following decade. Portable communication aids such as the LightWriter and the Talking Brooch were developed in the early 1970s with text‐to‐speech systems appearing in the late 1970s. More recently, developments in mobile technology and the explosion of social media are transforming the lives of disabled individuals who can access mainstream technology with little or no support. Similarly, the recent launch of the Global Public Inclusive Infrastructure (GPII)^1^—a web‐based resource supporting software engineers to develop accessible technology—demonstrates the degree to which technology developers have embraced the ideal of inclusive design to ensure that disabled people can benefit from technology. However, for individuals with complex communication needs (CCN) who use AAC, the successful adoption of technology, be it mainstream or specially designed AAC software, is fraught with challenges, resulting in low adoption and high abandonment of such technology.

Research has identified multiple reasons for low adoption and high abandonment of assistive and AAC technologies alike, including poor usability, high learning demands, a lack of professional expertise and difficulty in physical access (Murphy *et al*. 1996, Johnson *et al*. 2006, Hodge 2007, Baxter *et al*. 2012, Judge and Townend 2013). Individuals with CCN may have physical impairments which require different access methods such as switches or eye‐gaze systems to access AAC. Developing skills to master such technology requires a range of physical and cognitive competencies (Light and McNaughton 2014), which may take years to acquire, resulting in an inevitable focus on managing access and developing operational competencies, rather than using technology to achieve communication goals. While there is no doubt that AAC technology improves the quality of life for individuals with severe disabilities, the reality is that individuals with CCN seldom go beyond needs based (transactional) communication into extended (interactional) conversation (Waller 2006).

## Background

### AAC technology

AAC describes the strategies and techniques used to support communication for individuals who have little or no functional speech due to a physical and/or intellectual disability. At its simplest, AAC provides individuals with the means to make choices with early intervention identifying clear and consistent yes/no responses. At its most complex, users have to master complex operational skills in order to access AAC technologies.

AAC technology comes in many guises, depending on the communication needs of the individual. Communication without speech generation, using unaided AAC (e.g., gesture and eye pointing) and non‐technological aided AAC (e.g., symbol books and alphabet boards) is indispensable. However, SGDs provide users with a powerful bridge to independence. SDGs offer a route into education, employment, recreation and social inclusion. It has also been demonstrated that the ability to generate speech supports the development of language, natural speech and literacy (Schlosser 2003, Schlosser and Raghavendra 2004).

#### Speech generation

Speech output can be generated by recording audio, which is stored digitally and played back. Alternatively, text‐to‐speech synthesizers offer a range of voices and accents. SGDs use either digitized or text‐to‐speech output, or a combination of both. Whatever speech output mechanism is used, the challenge is to provide non‐speaking individuals with a means to generate conversation which is then output as speech. Literate users are able to generate text using physical or onscreen keyboards to type messages letter by letter, while emerging or pre‐literate communicators retrieve pre‐stored linguistic items (word, phrases and sentences) to generate messages. Retrieving pre‐stored items can increase the communication rate, reduce the physical effort of typing and provides access to linguistic items without the need to be literate.

#### Dedicated devices

Dedicated SGDs with static interfaces ranging from single or multiple message devices, provide simple access to speech output. Voice recorders can be programmed by recording a single or a series of short audio clips which can be replayed when the switch is activated. These devices are usually employed when supporting the development of cause and effect or to provide access to play, linking them to a toy or other activity. Waller and Black (2012) demonstrate how to extend the use of voice recorders to support *Storysharing* (Grove 2010) and provide guidelines on how individuals with complex needs can be involved in sharing personal experience. By anticipating the sequence in which a speaking communication partner will scaffold the sharing of an experience, i.e., engaging in interactive storytelling, the non‐speaking partner can take an active role in the interaction.

#### Visual scene displays (VSDs)

VSDs describe SGDs in which graphic scenes or photographs are used to provide context for embedded pre‐stored messages. Based on research in Dundee (Dye *et al*. 1998), VSDs (Blackstone 2004) are different to traditional dynamic display devices in that the displays are personalized and use highly contextual visual representations such as photographs and drawings. Recorded audio (e.g., speech/sound) or text is embedded under ‘hotspots’ within the VSD. When these hotspots are pressed, the pre‐stored audio or computer generated speech (using text‐to‐speech technology) is played. VSDs, in the form of apps on tablet devices, have been successfully applied to people with aphasia (Beukelman *et al*. 2015), children with CCN (Light and Drager 2007) and those with autism spectrum disorders (Chapin *et al*. 2018). Using approaches such as ‘Just‐in‐Time’ programming (Holyfield *et al*. 2018), practitioners are able to upload photographs into VSD systems and embed linguistic items under hotspots in real time, allowing communication partners to support interactions.

#### Dynamic display systems

Technologies which use dynamic screens provide multilevel message retrieval. Dynamic display systems (Waller 2009) mirror the use of communication books which are indexed on the first page. Each screen (or page) is usually organized as a grid. Cells (which can be labelled with icons, images or text) connect either to a linguistic item which is spoken on selection or to another page. Developers of dynamic screen AAC systems offer a variety of software solutions which provide a structured hierarchy of pages suitable for different stages of language acquisition. These pages are editable but require some training and effort to reprogram (Black *et al*. 2012).

#### Acceleration strategies

An alternative to multilevel message retrieval is to encode each linguistic item using a sequence of keystrokes. One such encoding system, Semantic Compaction (Baker 1982, 1987), provides an encoding strategy by which each linguistic item is retrieved using a sequence of up to three icons on static keyboards (consisting of as few as four icons up to a full keyboard of 144 icons). By learning the code sequences of a pre‐stored vocabulary, users can save significantly on keystrokes compared with letter‐by‐letter typing (Higginbotham 1992).

Word completion (suggested words, having typed the initial letters) and word prediction (suggests words which might follow the current word) can decrease the number of keystrokes required when typing (Swiffin *et al*. 1987, Higginbotham 1992). Using statistical and syntactic information, it is possible to predict probable words from what has already been typed. Although the increase in communication rate is at most 50% (Higginbotham 1992), word prediction/completion is of benefit to children learning to read and write; and dyslexic individuals who are able to recognize the target words they wish to type (Newell *et al*. 1992). Commercial word processors offer users the facility to store words or phrases under abbreviation sequences, e.g., ‘ph’ could expand into ‘telephone’. SGDs also incorporate such techniques, allowing users to store phrases such as ‘Best wishes, Annalu’ under a user‐defined sequence of keys, e.g., ‘BW’.

#### Mainstream technologies

The widespread adoption of social media and mobile technologies has made speech output technology, word prediction and image support available to disabled people on mainstream platforms. Features on mobile technology, e.g., photographs (Hanson *et al*. 2013) and access to the internet and use of speech output (Black *et al*. 2016), are being used to support communication. In contrast to debates in the 1990s, when researchers at Dundee were advised against developing AAC software on laptop computers as opposed to dedicated devices and questioned by therapists and teachers as to the ethics of word prediction (we were ‘putting words into the mouths of children’), clinicians are now finding themselves having to support mainstream technology (Black *et al*. 2016). The organic use of mainstream information technologies such as social media is taking the field into a new era (Light and McNaughton 2014, Hemsley *et al*. 2017).

### The problem of abandonment!

There is no doubt that AAC technologies have made an impact on the lives of people with CCN as can be attested by attending the annual Communication Matters Conference.^2^ AAC technologies do enable people to engage in education, work and social activities, but the effort to harness the potential of technology requires significant investment from family, professionals and ultimately the person who uses AAC. The potential of AAC technologies to transform lives is evident, but UK government reports on speech, language and communication, and education (Bercow 2008, Scottish Government 2012a, 2012b) highlight that even when assessment of highly specialized equipment is prescribed through local and regional specialist AT services, day‐to‐day use is dependent on support from family, educators, clinicians and/or care staff who have little or no training in supporting someone using AAC.

In reality, people who use AAC tend to rely on their low‐tech systems, e.g., unaided yes/no responses or paper‐based communication boards (Waller 2006, Judge and Townend 2013). Research (Murphy *et al*. 1996, Johnson *et al*. 2006, Hodge 2007) has identified multiple reasons for the low adoption and high abandonment of AAC technologies, including:

- poor usability;
- high learning demands;
- a lack of professional expertise; and
- difficulty in physical access.

Judge and Townend (2013) conducted a survey across 43 people who use AAC and 68 AAC professionals, and in‐depth interviews with 18 people who use AAC. Synthesis of the data identified the following factors as contributing to the abandonment of AAC technologies: ease of use (systems are not intuitive); reliability (systems often break down); technical support (systems require support not readily available); the voice and language of the device (systems are not always intelligible, the volume is not easily adjusted and systems do not offer personalization); the decision‐making process (lack of involvement in choosing technology); service delivery and access to services (difficulty in provision); family perceptions and support, staff training and partner competency (this being crucial to using systems); and communication rate (slow speeds of communication).

Organizations, such as Natspec^3^ (the UK membership association for organizations that offer specialist provision for students with learning difficulties and disabilities) and the Karten Network^4^ of information technology centres for disabled people in the UK, have called for both training and research for professionals working in the area of AT, of which AAC technologies form an important subset. Natspec projects such as the DART (Disseminating Assistive Roles and Technology) Project (Slaughter and Mobbs 2014) on AT provision in further education have proposed the role of the assistive technologist—a trained professional who can innovate advanced technology solutions which cross disciplinary boundaries of computing, engineering, psychology, education, social and healthcare. This is a new concept and a new professional cadre is required to realize it. There is a need for the training of assistive technologists which goes beyond the DART curriculum to include AAC training. Indeed, many online training and development resources already exist, e.g., the modules commissioned by the Scottish government based on the IPAACKS (Informing and Profiling AAC Knowledge and Skills)^5^ framework. However, there is a need to provide accredited training for a new profession who will work alongside other professionals to support and adapt the use of AT to meet the day‐to‐day needs of individuals, and those around them, in education, employment and in social settings.

The need for training in AT is paramount. However, the other significant factor influencing the adoption and use of AAC technology focuses on the design of these systems. Research into the design of AAC technologies at Dundee has, for many years, demonstrated how user‐centred design (UCD) is integral to the development of well‐designed ATs (Pullin 2009, Newell 2011). Prior (2011) suggests that, despite strong evidence that UCD leads to wider use of technology, end‐user involvement in the design of AAC technologies has been restricted to expert users (e.g., AAC professionals who work for the company). There is some evidence that disabled end users have had some role in giving feedback on the final products, but there is little or no evidence of industry employing UCD approaches which involve end users at all stages of development.

As outlined by Judge and Townend (2013), the reasons for the observed low adoption and high abandonment of AAC technologies are diverse, interrelated and complex. The design of technologies has been identified as part of this complexity in terms of ‘ease of use’ (what software engineers would recognize as ‘usability’) and the prohibitively slow rates of communication. Other device‐related issues contributing to abandonment such as the choice of voice, ability to use devices in sunlight and the ability to adjust the volume would be considered under the umbrella of functional software design requirements, while hardware reliability (e.g., power failures and lack of support) are non‐functional requirements.

## Design challenges

When considering why AAC technologies are abandoned from a design perspective, areas of challenge include: physical access to technology, prohibitively slow communication rates, poor literacy skills, vocabulary organization and training requirements, and the focus on needs‐based communication with little support for extended communication.

### Physical access

Perhaps the most researched area in AAC software design has been in the area of physical access to technology. People who use AAC technology often have severe physical impairments resulting in slow access due to poor hand function (von Tetzchner 2018). Individuals for whom direct access is not possible often use a scanning interface which is operated using a single switch (e.g., a head‐switch mounted to the wheelchair headrest); the interface iteratively highlights rows of icons until the row containing the right icon is reached, at which point the individual presses a switch—the system then iteratively highlights icons in the selected row until the switch is pressed again. Using this kind of interface, a user may only be able to speak one to two words per minute (wpm). However, recent developments in eye‐gaze technology have superseded the use of switch access, making direct selection a viable option for a wider group of users.

Commercial companies offer a wide range of high‐calibre access solutions, ranging from different types of single switches and joysticks to eye gaze. Despite the wide range of solutions, some individuals do not have reliable movement or eye control. Recent advances using brain–computer interfaces (Lazarou *et al*. 2018) and the potential to use bio‐feedback (Memarian *et al*. 2014) may offer viable access methods for individuals with conditions such as locked‐in‐syndrome and profound multiple disabilities which make it difficult to identify consistent and reliable responses needed to access technology.

Although physical access is primarily concerned with hardware, technology developers should take the design of the software interface into account as non‐technical support is required for daily use of such technology. For example, eye‐gaze systems use calibration software to maximize accuracy. Such software must be designed to be simple to use to reduce the possibility of abandonment.

### Communication rate

Communication rate remains a focal issue in the development of AAC technology, and yet communication rates remain prohibitively low. Even when individuals are able to speak, a pause of over 3 seconds results in an awkward silence, while pauses of around 0.5 seconds disrupt the flow of conversation (Jefferson 1989). In practice, communication using AAC tends to be prohibitively slow. Compared with speaking rates of between 125 and 185 wpm, aided communication rates fluctuate between 2 wpm (for scanning interfaces) and 8–10 words (for direct selection) (Swiffin *et al*. 1987). Current acceleration techniques, ranging from abbreviations to letter and word prediction, only increase rates up to 12–18 wpm and introduce usability issues such as having to scan word prediction lists visually (Higginbotham *et al*. 2012).

### Literacy skills, vocabulary organization and training requirements

It has been reported that up to 90% of individuals with congenital CCN struggle to acquire functional literacy (Foley and Wolter 2010), despite an acknowledgement that literacy acquisition is of critical importance for people with CCN (Mandak *et al*. 2018). In addition, people with acquired cognitive impairments, e.g., aphasia resulting from a stroke (cerebral vascular accident), also experience varying degrees of difficulty with both expressive and receptive written language. This restricts the user of AAC from generating their own vocabulary. Using icons and symbols, users are able to retrieve words and phrases which have been stored in their systems, usually by adults. While some individuals learn to retrieve linguistic items for interactive conversation, many resort to using one word answers or respond with yes and no signals. For those individuals who do master symbol based AAC technology, the reward is relatively fast access to speech output. It is impressive when interacting with an expert user of a Minspeak interface (Baker 1982, 1987) where each word only requires up to three icon sequences without the need to scan items visually on a screen. However, the learning effort needed to master such systems is substantial and takes commitment from the individual, instructors and family. From a software design perspective, one must question why all the effort is placed on the human actors while the technology only provides an electronic pigeon‐hole system in which linguistic items are stored for later retrieval.

The importance of introducing literacy programmes for all children with CCN, regardless of assumed intellectual impairment, has been championed by many practitioners (Smith 2005, Mandak *et al*. 2018). Despite evidence that speech output supports literacy learning (Schlosser 2003), AAC technology has yet to be designed to: (1) support ways in which pre/non‐literate users can access vocabulary not in their systems; and (2) bridge the transition between symbol based AAC and literacy in a progressive manner without having to change systems.

### Focus on needs‐based communication

Conversation does not exist for the sole purpose of making our physical needs known, but enables individuals to interact socially. Communication of social closeness and expression of personality is not restricted to isolated sentences but instead takes the form of a conversational narrative (Clarke and Clarke 1977). Narratives (personal stories) consist of anecdotes, jokes and experiences, and are used to promote social acceptance, social closeness and personality projection. Stories and anecdotes are particularly important as past experience and the ability to relate events is an essential part of a person's make‐up. Other people's view of us is largely dependent on relating and sharing common experiences.

Although some individuals who use SGDs do engage in extended conversation, many more tend to use one word or short sentence responses to questions (Light 1988, von Tetzchner and Martinsen 1996, Waller *et al*. 2001, Waller and O'Mara 2003), rather than initiating extended conversation. This passive style of interaction is compounded by the lack of topic initiation and story despite the fact that majority of conversation is characterized by sharing personal experience in the form of narrative (Cheepen 1988), People who use AAC, especially those with congenital disabilities, therefore rely on others to share personal information across transition boundaries (Balandin and Waller 2010).

One of the reasons for the lack of extended conversation is that design of most augmentative communication systems focus on the communication of needs and wants. The ability to engage in more complex types of communication, including the sharing of personal narrative, seldom develops in people who have grown up using AAC; the operational construction of narrative discourse is prohibitively slow and physically exhausting and without the experience and technological support to construct and use narrative pragmatically, the ability and desire to extend conversation remains elusive (Waller 2006). Some users of AAC technology do share personal experience, but this often occurs by giving a key word to a skilled partner who then co‐constructs the story with the non‐speaking partner (Waller and Black 2012).

The critical role of the ‘story guardian’ cannot be underestimated—family members tend to share the life stories of people who are unable to tell their own stories due to congenital, acquired or degenerative speech impairments. But what happens when those story guardians are unavailable or no longer around?

## Taking a pragmatic approach to the design of AAC systems

The aim of the Dundee AAC Research^6^ group is to utilize the power of computational linguistics and human–computer interaction to support more efficient and extended conversation. Over the past three decades, research at Dundee University has investigated how AAC technology can be designed to: improve communication rates, scaffold language development and support extended conversation. The examples below provide a flavour of how computational power, the use of natural language processing and the understanding of conversational pragmatics can result in systems which allow the user to focus on the linguistic and social aspects of communication rather than the operational and strategic aspects which currently have to be mastered first.

### Language play: access to rhyming puns—can a computer make a joke?

STANDUP, specifically designed to support children with CCN (Manurung *et al*. 2008, Waller *et al*. 2009), provided access to a specific form of interactive narrative—the telling of riddles. The punning riddles were generated using natural language generation technology, e.g., ‘What do you call a spicy missile? A hot shot.’ The children were able to generate a riddle and control the delivery of the riddle. Symbol support and different physical access methods (touch screen and switch accessed linear scanning) allowed users to access parts of the generated riddle phrases in a pragmatic way. The system not only produced the riddles, but also scaffolded their delivery so that the child could control the interaction by choosing to repeat the question (for clarification or to build tension) before revealing the answer. In addition, the complexity of the interface (the range of functionality ranged from a one‐button interface to generate any riddle to several buttons allowing for thematic riddle generation) and vocabulary was automatically determined by setting the child's user profile. A study (Waller *et al*. 2009) with children with cerebral palsy with emerging or assisted literacy skills used STANDUP over a period of 10 weeks. Video analysis revealed that all nine children spontaneously used the software without training on the interface. Video observation and summative interviews with staff indicated that participants were able to use the system to tell and re‐tell jokes to parents and others. Analysis of video and interviews supported researcher observations that the children and teachers had fun engaging with joke telling. There was some evidence that the children were aware that the jokes had not been pre‐stored by others and were therefore more motivated to engage in joke telling. Staff reported that this had a positive effect on their use of their standard AAC.

### Scaffolding language: narrative support—can a computer tell a story?

The ‘How was School Today?’ project (Tintarev *et al*. 2016) demonstrated how ‘data‐to‐text’ technology used commercially in fields such as the production of textual weather reports. By tagging people, objects and locations in a special school, the system generated simple phrases which formed a story event. Story events are identified when something is recorded as non‐routine (Reddington and Tintarev 2011), e.g., when there is a change in timetabled events or if staff have recorded a voice message during the event. Employing an ethnographic approach to understanding the context and user requirements, the system was designed to scaffold the telling of personal experience without the need for an adult to type a story into an AAC system. Black *et al*. (2012) present a detailed case study showing how a teenager with cerebral palsy was able to initiate and engage in extended conversation. Like STANDUP, the interface was designed to scaffold conversation in real time with minimal need for training. In particular, ‘How was School Today’ provides the pragmatic structure to introduce a narrative, control the pace of delivery and respond to partner interactions. A sequence of simple sentences are generated by the system and presented to the user as a ‘story‘. The user can speak each sentence in turn. By selecting left and right arrows the user can go back to a previous sentence or skip a sentence. Based on speech act theory, the user can choose to add emotion (‘evaluate‘) a sentence by selecting a ‘smiley’ or ‘frowny’ face. The system automatically generates a positive or negative emotional evaluation of the preceding utterance. For instance, after saying, ‘A visitor was there,’ a positive response might be ‘She was nice.’ The user thus has access to novel utterances without the need to compose these utterances. The rationale here, is to provide the child with the experience of initiating and controlling an interaction. Conversational analysis (Black *et al*. 2012) demonstrated the potential of such technology to scaffold a conversation enabling the child to engage in a more natural conversation, initiating, responding and evaluating how they felt about aspects of the narrative.

### Communication rate—can a computer support real‐time conversation?

Considering that good non‐disabled typists are able to achieve expressive writing speeds of only 27 wpm (Blackstone 1990), alternative strategies to traditional word for word typing are required to support users of AAC to engage in real‐time conversations. It has been suggested by Todman *et al*. (2008) that the only way to achieve communication rates which approach speaking rates of over 100 wpm is to utterance‐based systems (UBS). Research projects have demonstrated the potential of such systems to achieve rates of up to 64 wpm (Todman *et al*. 2008), compared with a theoretical speed of 25 wpm with the innovative letter/word prediction AAC system, Dasher (Ward and MacKay 2002). It is further advanced that well designed UBS which link pragmatic features and user goals can lead to faster communication without losing coherence. The TALK system (Todman *et al*. 2008) is based on a pragmatic model which sees the progression of a conversation as a series of gradual shifts of perspectives relating to the speaker, time (past, present, future) and event‐related information (what, where, who, how, and why). By changing perspective, the system can predict possible utterances. However, these systems rely on handcrafted sentences and the user needs to remember the conversational content and the location of this content.

## AAC today

The allure of technology as the ultimate AAC solution is as strong today as it was in the 1980s. The emergence of mobile technologies and social media over recent years has spawned a proliferation of AAC apps. High profile users of AAC—e.g. Professor Stephen Hawking; Lee Ridley (also known as Lost Voice Guy) who won the 2018 *Britain's Got Talent* competition with as a stand‐up comic; and Martin Pistorius (Pistorius 2013), award‐winning author—have raised public awareness of AAC. And yet, the issue of abandonment of AAC remains high, particularly when there is a poor fit between user and AAC systems, when AAC is not valued, and when there is a lack of support and training (Johnson *et al*. 2006).

Light and McNaughton (2012, 2014) reflected on the key changes in the field of AAC which further highlights the challenges of appropriate choice and use of AAC:

- The demographics of those who could benefit from AAC have changed in relation to the increase in the severity and variability of disability. Creer *et al*. (2016) report that 0.5% of the UK, 97% of whom have conditions such as dementia, Parkinson's disease, autism, learning disability, stroke, cerebral palsy, head injury, multiple sclerosis and motor neurone disease, could benefit from AAC. The increase of severity of physical disability and extended life expectancy suggests that AAC systems need to be more flexible and adaptable.
- There is a raised expectation for increased participation of disabled individuals in the community. Such participation means that systems need to support communication in a range of different settings and environments with the wider society. Again, AAC systems need to be more flexible and adaptable.
- Technology‐based AAC systems are no longer constrained to custom‐built systems—users of AAC need to access a wide variety of systems with the explosion of mobile platforms and social media. Studies (e.g., Hemsley and Murray 2015, Hemsley *et al*. 2017, Hynan *et al*. 2015) demonstrate the use and challenges faced by individuals who use AAC who wish to access mainstream information technology. Projects such as the GPII and the World Wide Web Consortium (W3C)^7^ illustrate the efforts of the computing industry to provide access for all. However, the language and communication requirements of people who use AAC are not easily accommodated.
- The scope of communication needs has broadened. There is now an awareness that AAC systems need to support complex communication. No longer is it sufficient to express wants and need, but there is an understanding that users of AAC systems need to go beyond transactional communication into extended conversation.

Light and McNaughton (2014) revisited Light's (1989) original definition of communicative competence for individuals who require AAC. Noting the changes discussed above, the demand on users to master linguistic, operational, social and strategic competencies is even more challenging. It can be argued that the design of AAC technology should address all these competencies. In reality, most AAC systems provide a sophisticated ‘pigeon hole’ system where the user is required: (1) to have a target message in mind (linguistic and social competencies); (2) to know what vocabulary is stored in the system (linguistic and operational competencies); (3) to match the target message with what the system can produce (strategic and linguistic competencies); (4) to recall where the target items are stored (operational competency); and (5) to plan and physically retrieve the item(s) (operational and strategic competencies).

Using the AAC systems of today demand high levels of competence with little or no support from the system, despite the potential of technology to provide adaptive support for users. Symbol communication systems are mostly static. Commercial systems have developed ranges of language packages which attempt to organize vocabulary in a logical way but these require intensive learning to engage in basic transactional communication. Systems do not automatically adapt in any way to mould to the way the user may organize language or to change as the user develops language and communication skills. Literacy‐based systems provide more linguistic support, but current AAC systems do not necessarily employ state of the art despite text entry technologies.

## Harnessing the potential of technology—dreaming the future

My research career has provided me with the privilege of dreaming the future with an amazing array of interdisciplinary colleagues and research students. Knowledge and understanding of UCD approaches and state‐of‐the‐art artificial intelligence have allowed us to develop and evaluate novel approaches to support and scaffold typical conversation. However, our dreams were often met with scepticism and accusations of being unethical. Having identified the need to support conversational narrative in the early 1990s (Waller and Newell 1997), clinicians were initially sceptical about people reusing conversational texts to share experiences. Similarly, UBS were viewed as impractical, due perhaps to the fact that users had to memorize and retrieve pre‐stored utterances without the support of a graphical interface. It was only during the ISAAC Research Symposium in 2000 that it suddenly dawned that audiences were trying to map new ideas of narrative on old paradigms. This led to the notion of training end users (clinicians, teachers, families and individuals who use AAC) to be more informed in terms of focusing on what technology should offer, not what they think technology can offer (Waller *et al*. 2005). With the advent of social media and the increasing use of artificial technologies, e.g., use of facial recognition, location tagging and automatic timelines, there is a general awareness that technology has the potential to provide a better user experience of AAC technologies.

Over 25 years of research focussing on improving the design of AAC technologies has demonstrated the potential: (1) to support extended conversation; (2) to reduce the operational/cognitive load on users; (3) to provide adaptive interfaces as users develop communicative competencies throughout their lives; and (4) to provide opportunities for language play without relying on others to expand vocabulary.

What is on the horizon? Despite, decades of research, the best AAC system remains the human dyad comprising speaking and non‐speaking partners who know each other intimately. Observations of such dyads demonstrate the importance of shared knowledge of past experience, topic identification, patterns of communication, individual preferences and nuances. More research needs to focus on such interactions or non‐typical conversations (e.g. Bloch and Beeke 2008) with a view to design systems which simulate the co‐construction on conversation. Research systems such as ‘How was School Today’? (Black *et al*. 2012) and TALK (Todman *et al*. 2008) demonstrate how systems can scaffold conversation. However, these systems require ongoing acquisition of personalized experiential language, leading to the concept of context‐aware AAC (e.g., Kane *et al*. 2012, Black *et al*. 2018). Another example of applying state of the art artificial intelligence to AAC is using object recognition to automatically label objects within a photograph (Hunter 2018).

Technology has such potential, but it is clear from the research on abandonment that recognizing the need to support communication partners and instructors as users of AAC systems is paramount. Projects such as the I‐ASC (Identifying Appropriate Symbol Communication Aids for Children who are Non‐Speaking: Enhancing Clinical Decision Making) Project^8^ and ethnographic studies in a special school (Norrie *et al*. 2018), are highlighting the importance of highly quality support. Even when technology is innovated in collaboration with a wide range of stakeholders and identified by multidisciplinary teams, the adoption of AAC technology depends on a knowledgeable support environment. AAC technology is not an appliance which is plugged in and switched on. Instead it is a tool requiring ongoing support, ideally from assistive technologists working with educators and care staff to ensure that the true potential of AAC technology is realized!

## Notes

1 For the Global Public Inclusive Infrastructure (GPII), see https://goo.gl/esjy21/

2 For the UK Chapter of ISAAC, see http://www.communicationmatters.org.uk/

3 For Natspec, see https://natspec.org.uk/

4 For the Karten Network, see https://karten-network.org.uk/

5 For IPAACKS and training modules, see http://www.nowhearme.co.uk/information-for-professionals/

6 For the Dundee AAC Research Group, see http://www.aac.dundee.ac.uk/

7 For the World Wide Web Consortium (W3C) see https://www.w3.org/

8 For the I‐ASC Project, see http://www.i-asc.org.uk/
